# Associates of Perceived Quality of Life in Chinese Older Adults Living With Cognitive Impairment

**DOI:** 10.1093/geroni/igab046.941

**Published:** 2021-12-17

**Authors:** Xiang Gao

**Affiliations:** Huazhong University of Science and Technology, Wuhan, Hubei, China (People's Republic)

## Abstract

This study examined perceived quality of life in Chinese older adults living with cognitive impairment in a group of urban Chinese older adults and explore its associations with caregivers’ characteristics. Questionnaires were administered in person to 300 caregiver-care recipient dyads from three urban communities in mainland China in 2019. The 40-item Alzheimer’s Disease-related Quality of Life tool asked caregiver respondents to indicate care recipients’ life conditions. Higher levels of caregiving burden (β = -0.19, p < 0.01) and more depressive symptoms (β = -0.19, p < 0.01) amongst caregivers were significantly associated with lower quality of life of care recipients. The results suggested that reducing caregivers’ burden and depressive symptoms are essential to promote quality of life of care recipients. Formal support from health professionals, service organizations, and communities are urgently called for to promote the wellbeing of Chinese families affected by cognitive impairment.

